# Greenness assessment and phototoxicity of rose bengal and methylene blue on immature aquatic stages of malaria vector *Anopheles pharoensis*

**DOI:** 10.1038/s41598-025-03519-1

**Published:** 2025-05-26

**Authors:** Ahmed Z. I. Shehata, Ahmed A. El-Mehdawy, Mohammed A. Mahmoud, Lina A. Abou El-Khashab, Ahmed M. Saleh, Ahmed N. G. Abdel-Aziz, Nader A. Bakr, Heba F. Abd-Elkhalek, Mohamed A. M. El-Tabakh

**Affiliations:** 1 Department of Zoology, Faculty of Science, Al-Azhar University, 11651 Cairo, Egypt; 2 Lecturer of Molecular biology - zoology and Entomology department, Faculty of Science, Al-Azhar University, Cairo, 11884 Egypt; 3 Department of Pharmaceutical Chemistry, Faculty of Pharmacy, Horus University, New Damietta, Egypt; 4 Consultant Medical Entomologist at the Public Health Department, Jazan Municipality, Jazan, Saudi Arabia; 5 Entomology Department, Faculty of Science, Benha University, Benha, Egypt

**Keywords:** *Anopheles pharoensis*, Complex GAPI, Larvicidal, Methylene blue, Photosensitizers, Rose bengal, Biological techniques, Biotechnology, Drug discovery, Molecular biology

## Abstract

This study systematically evaluated the efficiency of rose bengal and methylene blue as photosensitizers against the immature aquatic stages of *Anopheles pharoensis*. Genetic identification using the COI partial sequence confirmed the species, and the obtained sequence was submitted to GenBank (Accession No. PQ346929). Both photosensitizers exhibited 100% mortality in larvae I within 24 h at their highest concentrations, demonstrating strong biocidal activity. LC_50_ values for rose bengal increased from 1.50 ppm (24 h) and 1.34 ppm (48 h) in larvae I to 3.83 ppm (24 h) and 3.12 ppm (48 h) in pupae. Similarly, methylene blue showed LC_50_ values rising from 1.14 ppm (24 h) and 0.90 ppm (48 h) in larvae I to 2.91 ppm (24 h) and 2.51 ppm (48 h) in pupae, indicating stage-dependent susceptibility. Enzymatic responses revealed a progressive increase in acetylcholinesterase (AChE) and glutathione S-transferase (GST) activity in the developmental stage, suggesting a physiological adaptation to the photosensitizers. Molecular docking against the AChE protein (PDB ID: 6xyu) confirmed insecticidal bioactivity, with methylene blue exhibiting superior binding affinity, aligning with the in-vitro larvicidal results. Furthermore, a Complex GAPI assessment confirmed the environmental sustainability of both photosensitizers, supporting their potential as eco-friendly alternatives for mosquito control. The use of Complex GAPI in assessing the environmental sustainability of photosensitizers in mosquito control represents a novel approach in the field of integrated pest management. This advancement not only aligns with the principles of green chemistry but also addresses the growing need for sustainable alternatives to traditional chemical insecticides. These findings highlight the feasibility of utilizing light-activated photosensitizers for sustainable vector management.

## Introduction

Mosquito-borne diseases are the most relevant arthropods in public health, especially Anopheles genera because of their responsibility in transmitting different human and animal diseases as malaria^1^. Malaria is a parasitic disease caused by Plasmodium protozoa and infects more than 240 million people worldwide causing more than 625 thousand deaths in 2021^2^. To eliminate the spread of malaria, several strategies have been applied to control the prevalence of different *Anopheles Sp.*^3^. Different immature aquatic stages of *Anopheles Sp.* were usually targeted by synthetic chemical insecticides, but developing new control alternatives, which are more efficient, safe, and eco-friendly considered acceptable proper and urgent replacements to avoid the hazards of synthetic chemical insecticides^4,5^.

Photosensitizing agents, which are activated by sunlight, attracted more attention as a new generation of insecticide that is highly efficient and environmentally safe due to its rapid photodegradation in the visible light^6^. Photo-insecticides have been used in agriculture against crop pests^7^. Photodynamic therapy (PDT) is used clinically to treat a wide range of medical conditions, including psoriasis, and atherosclerosis and has shown some efficacy in anti-viral treatments, including herpes. It also treats malignant cancers including head and neck, lung, bladder, and particular skin^8^. Also, this technology has been tested for the treatment of prostate cancer in a dog model and human prostate cancer patients^9,10^. In addition, PDT is recognized as a treatment strategy that is both minimally invasive and minimally toxic.

Evaluating the greenness of the method is essential for advancing environmental sustainability. This assessment encompasses a rigorous examination of several critical parameters, including solvent consumption, waste production, energy requirements, and process efficiency^11^. By applying green chemistry principles, this evaluation provides a quantitative measure of a method’s ecological impact, thereby facilitating the development of more sustainable practices and promoting adherence to environmental standards. The complex GAPI tool represents a significant advancement in the assessment of method greenness, providing a comprehensive and systematic approach for evaluation^12^.

The current study aimed at evaluating the impact of two major photosensitizers, rose bengal and methylene blue against different aquatic immature stages of the malarial vector, *Anopheles pharoensis.*

## Materials and methods

### ***Anopheles pharoensis*** colony

Larvae of *Anopheles pharoensis* were obtained from Medical Entomology institution, Dokki, Giza, Egypt and reared for several generations under controlled conditions of temperature (25 ± 2 °C), relative humidity (70 ± 10%) and photoperiod (12 L:12D) following a standard rearing procedure described by **Hassanain** et al.^13^ to provide immature stages needed for the bioassay.

### Genetic identification of ***Aedes aegypti*** larvae

#### Extraction of DNA from mosquito larvae

Small pieces of the *A. pharoensis* specimens were inserted in 1.5 µL Eppendorf tubes for study. The PureLink^®^ Genomic DNA Kits were used for DNA extraction (Invitrogen, Waltham, Massachusetts, USA). In summary, 180–250 µL of tissue lysis buffer was added to each sample, and 10 µL of proteinase K was added to each 180 µL of tissue lysis solution. Then, for 4 h, the combination was kept at 56 degrees Celsius. The supernatant was transferred to a fresh tube as directed by the manufacturer (Invitrogen; Waltham, Massachusetts; USA). Following the addition of 200 µL of ethanol and 200 µL of Lysis/Binding Buffer, the lysate was vertexin. The solution was then placed in a spin column and centrifuged at 10,000 xg for one minute. DNA was eluted in 50 µL of elution solution after two washes with wash buffers and then kept at -20 °C.

#### Polymerase chain reaction (PCR)

LCO1490 (5’-GGTCAACAAATCATAAAGATATTGG-3’) and LCO1490-R (5’-TAA ACT TCA GGG TGA CCA AAA AAT CA-3’) were used as the forward and reverse primers, respectively. A total of 50 µl was used for the PCR amplification process, with 25 µl used for the 2X master mix solution (i-Taq, iNtRON, Seongnam, Korea), 2 µl each of the 0.2 M primers, 4 µl of template DNA, 2 µl of BSA at 0.2 mg/mL, and 14.5 µl of nuclease-free water. First, tick DNA was denatured at 95 degrees Celsius for 10 min, then it was subjected to 40 cycles of denaturation at 95 degrees Celsius for 1 min, annealing at 46 degrees Celsius for 1 min, and extension at 72 degrees Celsius for 1 min. The last 10-minute extension was carried out at 72 degrees. The amplified DNA was seen on a 1% agarose gel stained with ethidium bromide and examined under a transilluminator to determine the PCR product’s quality and quantity (U.V. transilluminator, Spectroline, Westbury, USA).

#### Sequence analysis

Using a Macrogen reagent, the PCR products were isolated and purified (Seoul, Korea). Nucleotide sequences of *A. pharoensis* COI were aligned following single-strand DNA sequencing.

### Assessment of photosensitizer activity

Rose bengal and Methylene blue (purchased from Sigma-Aldrich Company, Egypt) with molecular weights of 1017.64 and 319.85, were tested against *An. pharoensis* different immature stages. Different concentrations of each photosensitizer were prepared using distilled water for the application. Sunlight was used as a light source for the activation of tested photosensitizers. Incubation time was detected according to the method described by El-Mehdawy et al.^14^.

Twenty-five individuals of each immature stage were deprived of food for six hours and then placed in 300 ml beakers containing 250 ml of different concentrations from each photosensitizer. Then, larval stages were allowed to feed on a small piece of bread for six hours in the dark, and subsequently tested stages were washed extensively to remove the excess photosensitizers and transferred into 200 ml of clean distilled water for the irradiation process. Control groups represented in different concentrations from all tested photosensitizers in the dark. The irradiation process was carried out using sunlight for 20 min. From 12.00 to 12.20 am along with control groups (photosensitizers’ free) to assess the impact of sunlight alone on immature stages. Mortality was recorded right after radiation and after 24 and 48 h. Three replicates of each test were usually used^14,15^.

### Enzymatic measurements

AChE and GST are key biomarkers in toxicological studies. AChE is used to assess neurotoxic effects, while GST helps evaluate detoxification and oxidative stress. Together, they provide insights into the physiological effects of photosensitizers, making them essential for understanding their environmental and biological impacts. For the quantification of AChE and GST, 10 ml solutions of 0.1 M phosphate buffer, pH 7.5 (KH2PO4 - NaOH), incorporating 1% Triton X-100, 1% ethanol, and 1% Triton X-100, respectively, were utilized to homogenize three batches of the specified immature stage (derived from each assessed LC50). The Hereaeus Labofuge 400R, manufactured by Kendro Laboratory Products GmbH, Germany, was utilized to centrifuge the homogenates for 60 min at 4 °C and 15,000 x g. The resulting supernatant underwent an in vitro Ache (U/L) inhibition experiment without additional purification^16^. The GST activity (U/g tissue) was assessed using spectrophotometric measurements of supernatant aliquots, following the methodology outlined in the accompanying brochure for spectrophotometric testing^17^.

### Molecular docking test

To study the binding mechanism and interactions between the two complexes and the AChE enzyme (PDB ID: 6xyu), molecular docking studies were performed using MOE 2015 software. The 3D models of AChE were retrieved from the Protein Data Bank (PDB ID: 6xyu) at http://www.rcsb.org.pdb. The 3D structures of the ligands and the most potent complexes were created using Chem Draws 18.0 and stored as MDL molfiles. The greatest score went to the chemical that had the lowest binding affinity value.

### Statistical analysis

All data were analyzed using SPSS (Version 22) and Minitab (Version 14) statistical software. The normality of data distribution was tested using the Shapiro-Wilk and Kolmogorov-Smirnov tests. For non-normally distributed data, Arcsine square root transformation was applied to standardize mortality percentages. Probit analysis was conducted to determine the LC_50_ and LC_90_ values along with their 95% confidence intervals (CIs) for each photosensitizer. One-way ANOVA, followed by Tukey’s post hoc test, was used to compare the mortality rates and enzymatic activities among different treatment groups. A Chi-square (χ²) test was applied to assess significant variations in mortality between treatment and control groups. The significance level was set at *P* < 0.05. Data are presented as mean ± standard deviation (SD), and all experiments were conducted in triplicates to ensure reproducibility. Data visualization was performed using R Studio (Version 2022.02.4) to generate graphs and statistical plots.

## Results

**Fig. 1:**
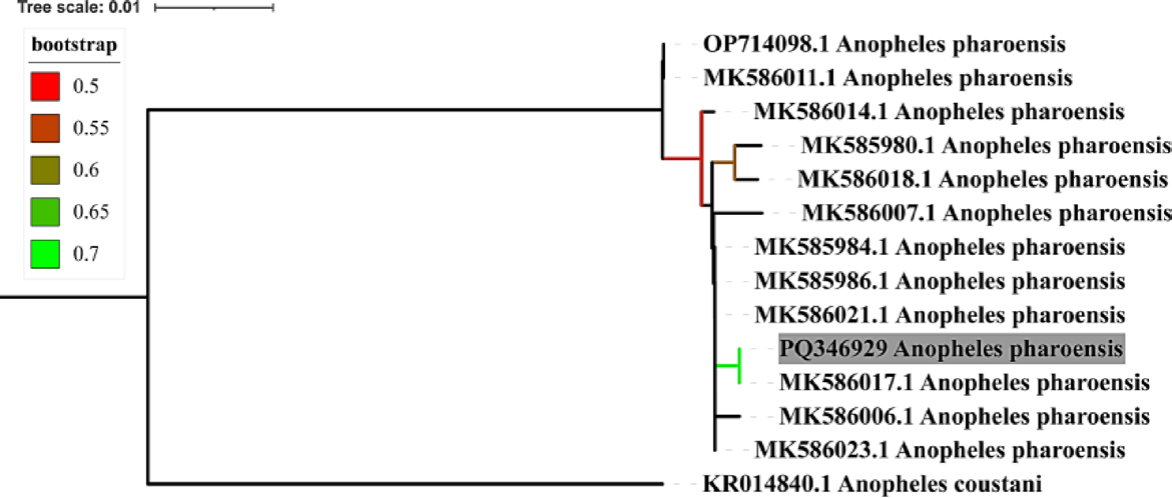
Neighbor joining phylogenetic tree.

*Anopheles pharoensis* has been genetically characterized using the partial sequencing of COI. The recently acquired sequence has been deposited in the gene bank and assigned the accession number PQ346929 (Fig. 1).

**Fig. 2:**
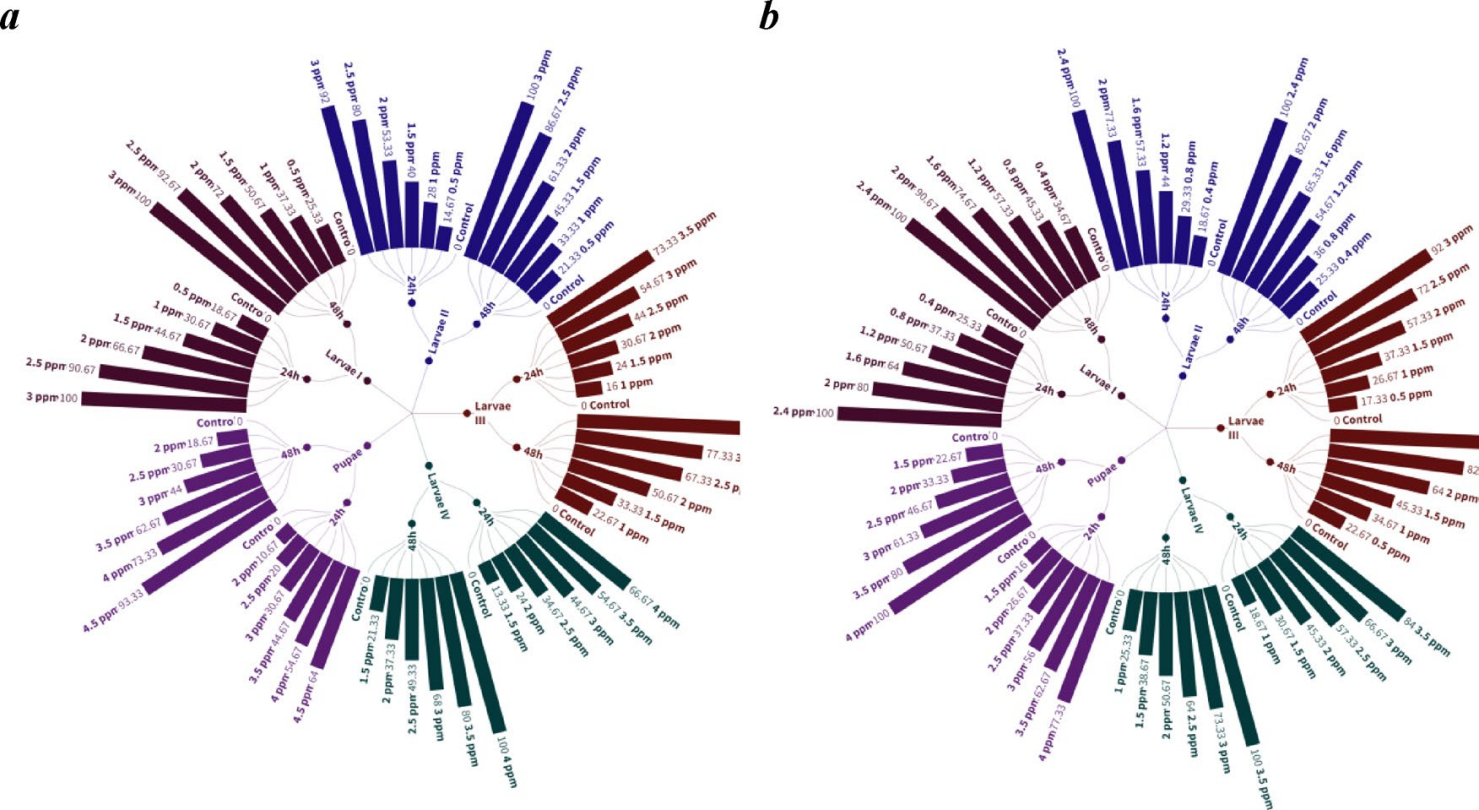
Mortality % of (**a**) Rose bengal and (**b**) Methylene blue against *Anopheles pharoensis* at different immature aquatic stages.

Mortality rates rise significantly with higher concentrations and prolonged exposure. Notably, at the highest doses, both photosensitizers induce 100.0% mortality in first-instar larvae within 24 h, indicating their strong biocidal properties. This high level of efficacy is maintained through the subsequent larval stages, although a slight decline in mortality is observed as the concentration decreases. At the pupal stage, both photosensitizers remain effective, but higher concentrations are necessary to achieve sustained mortality. Specifically, rose Bengal achieves a peak mortality rate of 93.33%, while methylene blue reaches 100.0% mortality at its highest concentrations within 48 h **(**Table 1 & Fig. 2).

**Table 1:** Mortality % of *Anopheles pharoensis* immature aquatic stages caused by tested photosensitizers.

Target stage	Rose Bengal			Methylene blue		
	Conc. (ppm) (dist. water)	24 h	48 h	Conc. (ppm)	24 h	48 h
Larvae I	3.0	100 ± 0^a^	100 ± 0^a^	2.4	100 ± 0^a^	100 ± 0^a^
	2.5	90.66 ± 3.77^a^	92.66 ± 7.71^a^	2.0	80 ± 3.26^b^	90.66 ± 1.88^b^
	2.0	66.66 ± 4.98^b^	72 ± 3.26^b^	1.6	64 ± 3.26^c^	74.66 ± 1.88^c^
	1.5	44.66 ± 0.94^c^	50.66 ± 1.88^c^	1.2	50.66 ± 1.88^d^	57.33 ± 1.88^d^
	1.0	30.66 ± 1.88^d^	37.33 ± 1.88^d^	0.8	37.33 ± 1.88^e^	45.33 ± 1.88^e^
	0.5	18.66 ± 3.77^e^	25.33 ± 3.77^d^	0.4	25.33 ± 3.77^f^	34.66 ± 1.88^f^
	Control	0^f^	0^e^	Control	0^g^	0^g^
Larvae II	3.0	92 ± 3.26^a^	100 ± 0^a^	2.4	100 ± 0^a^	100 ± 0^a^
	2.5	80 ± 3.26^b^	86.66 ± 1.88^b^	2.0	77.33 ± 3.77^b^	82.66 ± 4.98^b^
	2.0	53.33 ± 1.88^c^	61.33 ± 1.88^c^	1.6	57.33 ± 1.88^c^	65.33 ± 1.88^c^
	1.5	40 ± 3.26^d^	45.33 ± 1.88^d^	1.2	44 ± 3.26^d^	54.66 ± 1.88^d^
	1.0	28 ± 3.26^e^	33.33 ± 3.77^e^	0.8	29.33 ± 1.88^e^	36 ± 2.82^e^
	0.5	14.66 ± 1.88^f^	21.33 ± 1.88^f^	0.4	18.66 ± 1.88^f^	25.33 ± 1.88^f^
	Control	0^g^	0^g^	Control	0^g^	0^g^
Larvae III	3.5	73.33 ± 1.88^a^	100 ± 0^a^	3.0	92 ± 3.26^a^	100 ± 0^a^
	3.0	54.66 ± 1.88^b^	77.33 ± 1.88^b^	2.5	72 ± 3.26^b^	82.66 ± 4.98^b^
	2.5	44 ± 3.26^c^	67.33 ± 0.94^c^	2.0	57.33 ± 1.88^c^	64 ± 3.26^c^
	2.0	30.66 ± 1.88^d^	50.66 ± 1.88^d^	1.5	37.33 ± 1.88^d^	45.33 ± 1.88^d^
	1.5	24 ± 3.26^d^	33.33 ± 1.88^e^	1.0	26.66 ± 1.88^e^	34.66 ± 1.88^e^
	1.0	16 ± 0^e^	22.66 ± 1.88^f^	0.5	17.33 ± 1.88^f^	22.66 ± 1.88^f^
	Control	0^f^	0^g^	Control	0^g^	0^g^
Larvae IV	4.0	66.66 ± 1.88^a^	100 ± 0^a^	3.5	84 ± 3.26^a^	100 ± 0^a^
	3.5	54.66 ± 3.77^b^	80 ± 3.26^b^	3.0	66.66 ± 1.88^b^	73.33 ± 1.88^b^
	3.0	44.66 ± 0.94^c^	68 ± 3.26^c^	2.5	57.33 ± 1.88^c^	64 ± 3.26^c^
	2.5	34.66 ± 1.88^d^	49.33 ± 1.88^d^	2.0	45.33 ± 1.88^d^	50.66 ± 1.88^d^
	2.0	24 ± 3.26^e^	37.33 ± 1.88^e^	1.5	30.66 ± 1.88^e^	38.66 ± 1.88^e^
	1.5	13.33 ± 1.88^f^	21.33 ± 1.88^f^	1.0	18.66 ± 1.88^f^	25.33 ± 1.88^f^
	Control	0^g^	0^g^	Control	0^g^	0^g^
Pupae	4.5	64 ± 3.26^a^	93.33 ± 1.88^a^	4.0	77.33 ± 1.88^a^	100 ± 0^a^
	4.0	54.66 ± 1.88^b^	73.33 ± 1.88^b^	3.5	62.66 ± 1.88^b^	80 ± 3.26^b^
	3.5	44.66 ± 0.94^c^	62.66 ± 1.88^c^	3.0	56 ± 2.82^b^	61.33 ± 1.88^c^
	3.0	30.66 ± 1.88^d^	44 ± 3.26^d^	2.5	37.33 ± 1.88^c^	46.66 ± 1.88^d^
	2.5	20 ± 3.26^e^	30.66 ± 1.88^e^	2.0	26.66 ± 1.88^d^	33.33 ± 1.88^e^
	2.0	10.66 ± 1.88^f^	18.66 ± 1.88^f^	1.5	16 ± 3.26^e^	22.66 ± 3.77^f^
	Control	0^g^	0^g^	Control	0^f^	0^g^

The study clearly demonstrates a progressive increase in the LC_50_ values for rose bengal from larvae I to pupae, starting at 1.50 ppm (24 h) and 1.34 ppm (48 h) for first-instar larvae, and peaking at 3.83 ppm (24 h) and 3.12 ppm (48 h) for pupae. This upward trend suggests a reduced sensitivity of Anopheles pharoensis to rose bengal as the organism matures, requiring higher concentrations to achieve the same mortality rate in later developmental stages. Similarly, methylene blue follows a comparable pattern, with the lowest LC_50_ values recorded for larvae I at 1.14 ppm (24 h) and 0.90 ppm (48 h), steadily increasing through the developmental stages and reaching a peak for pupae at 2.91 ppm (24 h) and 2.51 ppm (48 h). Based on these findings, methylene blue appears to be more effective than rose bengal in terms of its overall toxicity across all stages. **(**Table 2**)**.

**Table 2:** The LC_50_ values (ppm) of tested photosensitizers against *Anopheles pharoensis* in different immature aquatic stages.

Photosensitizers	Target	LC_50_ (LCL-UCL) (ppm)		*χ2*	
		24 h	48 h	24 h	48 h
Rose Bengal	Larvae I	1.50 (1.39–1.61)	1.34 (1.15–1.53)	1.17^N**S**^	0.82^N**S**^
	Larvae II	1.71 (1.61–1.80)	1.50 (1.33–1.68)	1.18 ^NS^	0.93^N**S**^
	Larvae III	2.68 (2.42–2.93)	1.97 (1.90–2.04)	0.68^N**S**^	0.86^N**S**^
	Larvae IV	3.25 (3.02–3.47)	2.45 (2.30–2.60)	0.64^N**S**^	1.07^N**S**^
	Pupae	3.83 (3.63–4.03)	3.12 (3.01–3.23)	0.47^N**S**^	1.32^N**S**^
Methylene Blue	Larvae I	1.14 (1.07–1.21)	0.90 (0.88–0.92)	1.04^N**S**^	0.40^N**S**^
	Larvae II	1.29 (1.27–1.32)	1.12 (1.07–1.17)	0.54^N**S**^	0.86^N**S**^
	Larvae III	1.74 (1.56–1.91)	1.48 (1.27–1.69)	0.15^N**S**^	1.04^N**S**^
	Larvae IV	2.23 (2.09–2.38)	1.93 (1.78–2.08)	0.61^N**S**^	0.89^N**S**^
	Pupae	2.91 (2.71–3.12)	2.51 (2.40–2.62)	0.96^N**S**^	1.18^N**S**^

LC_50_ Half Lethal Concentrations; ppm part per million;* LCL* Lower confidence limit, *UCL* upper confidence limit; χ2 Chi-square value; NS non-significant.

For rose bengal, acetylcholinesterase (AChE) activity progressively increases from first-instar larvae to pupae, suggesting either a compensatory mechanism to counteract the photosensitizer’s effects or a stage-specific sensitivity to enzymatic activity. Specifically, AChE activity starts at 4.86 ± 0.07 in larvae I and peaks at 6.21 ± 0.17 in pupae after 24 h. A similar trend is observed for glutathione S-transferase (GST), with activity rising from 0.53 ± 0.02 in larvae I to 1.24 ± 0.06 in pupae, indicating a higher detoxification effort in later stages.

**Fig. 3:**
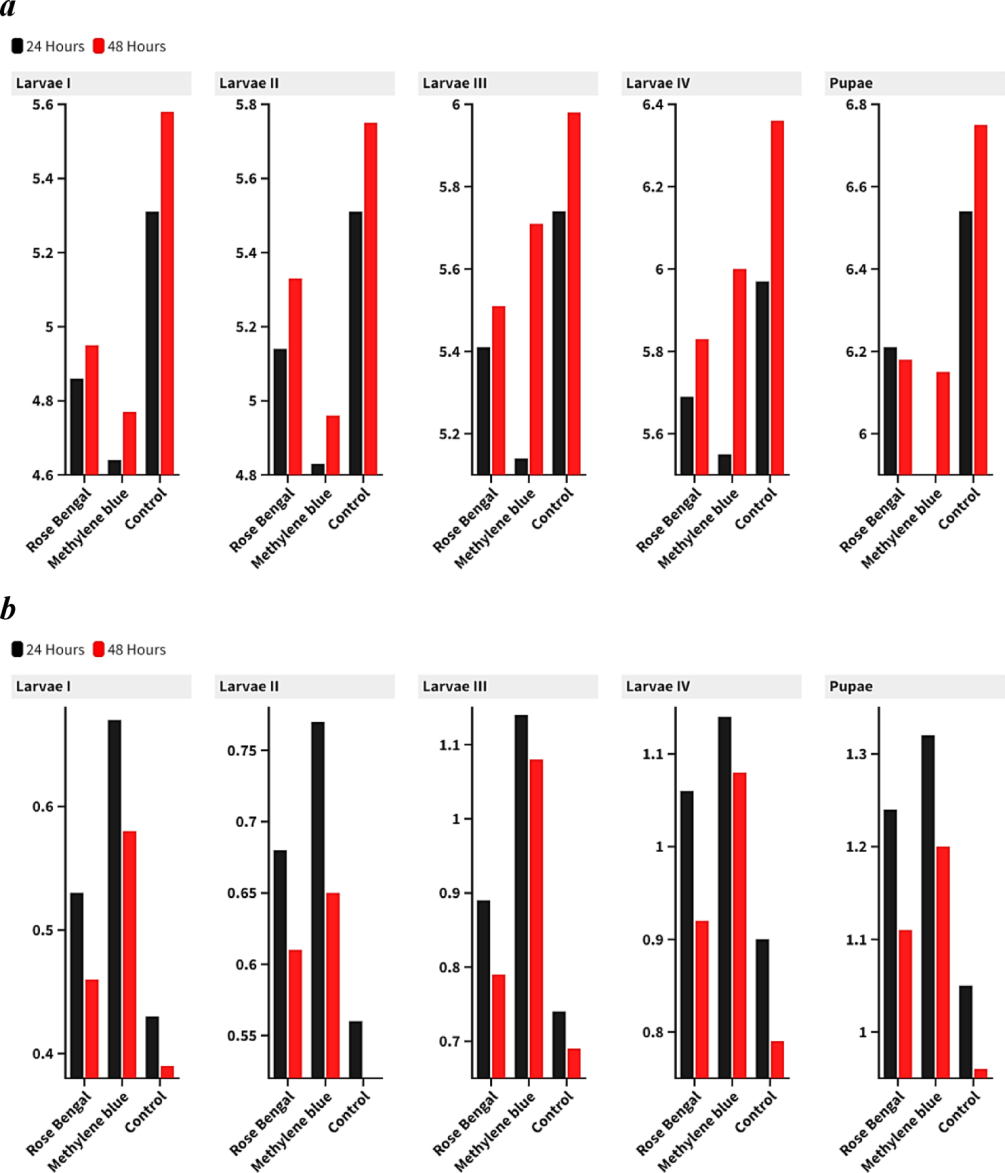
Enzymatic activity (**a**) AChE (U/l) and (**b**) GST (U/g tissue) against *Anopheles pharoensis* at different immature aquatic stages.

Methylene blue induces a comparable response, with both AChE and GST activity increasing from early larval stages to pupae. AChE activity rises from 4.64 ± 0.07 in larvae I to 5.90 ± 0.07 in pupae after 24 h, while GST activity increases from 0.67 ± 0.04 to 1.32 ± 0.04, respectively. This progressive rise suggests that as the mosquito matures, its enzymatic systems become more efficient at mitigating the oxidative stress or neurotoxic effects induced by methylene blue. Importantly, both photosensitizers cause significant differences (*P* < 0.05) when compared to the control groups, with treated groups exhibiting higher enzyme activities than those observed in the controls **(**Table 3 & Fig. 3).

**Table 3:** Enzymatic activity of tested photosensitizers against *Anopheles pharoensis* at different immature aquatic stages.

Photosensitizers	Larval stage	Acetylcholinesterase (AChE)		Glutathione-S-transferase (GST)	
		24 h	48 h	24 h	48 h
Rose Bengal	Larvae I	4.86 ± 0.07^e^	4.95 ± 0.08^c^	0.53 ± 0.02^e^	0.46 ± 0.04^e^
	Larvae II	5.14 ± 0.08^d^	5.33 ± 0.04b^c^	0.68 ± 0.06^d^	0.61 ± 0.03^d^
	Larvae III	5.41 ± 0.08^c^	5.51 ± 0.11ab^c^	0.89 ± 0.02^c^	0.79 ± 0.03^c^
	Larvae IV	5.69 ± 0.06^b^	5.83 ± 0.06a^b^	1.06 ± 0.06^b^	0.92 ± 0.04^b^
	Pupae	6.21 ± 0.17^a^	6.18 ± 0.64^a^	1.24 ± 0.06^a^	1.11 ± 0.06^a^
Methylene blue	Larvae I	4.64 ± 0.07^e^	4.77 ± 0.06^c^	0.67 ± 0.04^d^	0.58 ± 0.04^d^
	Larvae II	4.83 ± 0.07^d^	4.96 ± 0.08^c^	0.77 ± 0.05^d^	0.65 ± 0.02^d^
	Larvae III	5.14 ± 0.05^c^	5.71 ± 0.05^b^	1.14 ± 0.05^c^	1.08 ± 0.04^c^
	Larvae IV	5.55 ± 0.08^b^	6.0 ± 0.10^a^	1.14 ± 0.05^b^	1.08 ± 0.04^b^
	Pupae	5.90 ± 0.07^a^	6.15 ± 0.07^a^	1.32 ± 0.04^a^	1.20 ± 0.02^a^
Control (dist. water)	Larvae I	5.31 ± 0.09^d^	5.58 ± 0.10^d^	0.43 ± 0.05^d^	0.39 ± 0.03^d^
	Larvae II	5.51 ± 0.07^d^	5.75 ± 0.05^d^	0.56 ± 0.06^d^	0.52 ± 0.05^c^
	Larvae III	5.74 ± 0.11^c^	5.98 ± 0.04^c^	0.74 ± 0.05^c^	0.69 ± 0.04^b^
	Larvae IV	5.97 ± 0.06^b^	6.36 ± 0.10^b^	0.90 ± 0.05^b^	0.79 ± 0.04^b^
	Pupae	6.54 ± 0.07^a^	6.75 ± 0.07^a^	1.05 ± 0.07^a^	0.96 ± 0.06^a^

**Fig. 4:**
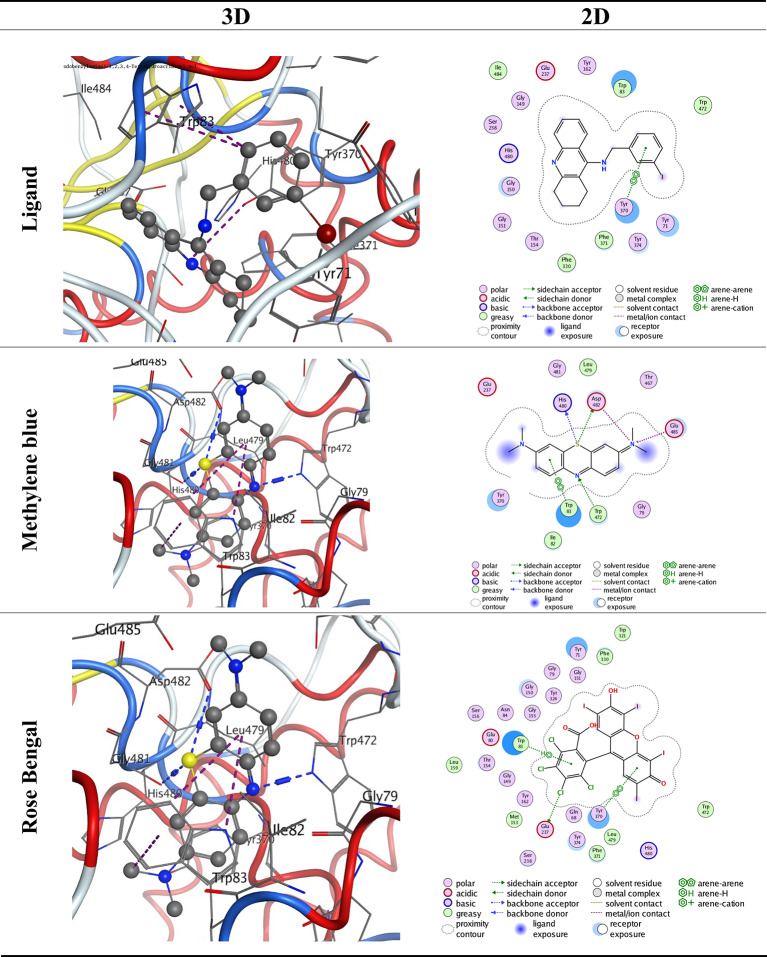
2D & 3D interaction of methylene blue and rose bengal complexes against the active site of AChE (PDB ID: 6xyu).

Molecular docking was performed on both methylene blue and rose Bengal, utilizing the target molecules against AChE (PDB ID: 6xyu) via MOE 2015. The energy scores of the molecules (Table 4) were ascertained to be -7.4948 and − 6.9603 kcal/mol for the Methylene blue and Rose Bengal complexes, respectively, surpassing the control ligand (9-(3-Iodobenzylamino)-1,2,3,4-Tetrahydroacridine) at -6.5522 kcal/mol. The intensity of the contact correlates positively with the magnitude of the negative binding energy. The interaction occurred in the sequence of methylene blue followed by rose Bengal complexes. These results align with experiments on insecticidal activity. Figures (4) illustrates the comprehensive bonding interactions of the pertinent amino acid residues in the AChE (PDB ID: 6xyu) with the docked molecules.

**Table 4:** Docking results inside ache (PDB ID: 6xyu) active spots, arranged from the negatively highest to the lowest score.

	Ligand	Receptor	Interaction	Distance (in Ao from main residue)	E (Kcal/mol)	S (Kcal/mol)
Ligand	6-ring	TYR 370	pi-pi	3.9	0	-6.5522
Methylene Blue	S 18	HIS 480	H-donor	3.28	-0.9	-7.4948
	S 18	ASP 482	H-donor	3.35	-1.4	
	N 28	TRP 472	H-acceptor	2.79	-2.2	
	N 1	ASP 482	ionic	3.96	-0.6	
	N 1	GLU 485	ionic	2.98	-4.6	
	6-ring	83	pi-pi	3.42	0	
Rose Bengal	CL 35	GLU 237	H-donor	3.32	-0.7	-6.9603
	6-ring	TRP 83	pi-H	4.57	-0.6	
	6-ring	TYR 370	pi-pi	3.76	0	

### Assessment of method greenness

**Fig. 5:**
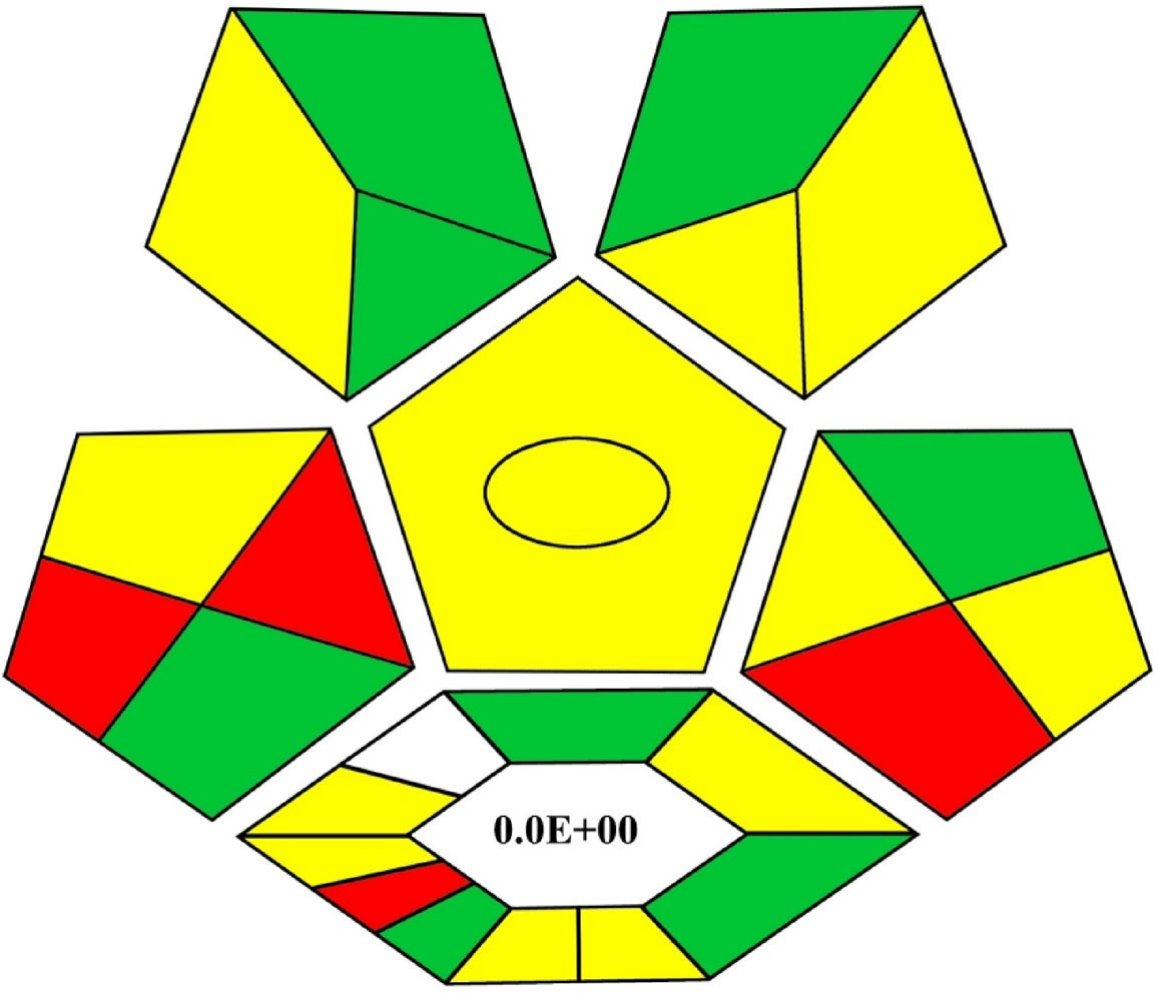
the complex GAPI metric.

The integration of green chemistry principles represents a significant advancement in promoting sustainability. The Complex GAPI framework has emerged recently as a critical tool for assessing the greenness of methods, which evaluates multiple dimensions of sustainability, including solvent use, waste generation, energy consumption and method efficiency. This method is evaluated as eco-friendly and environmentally sustainable based on the Complex GAPI metric as shown in Figure 5.

## Discussion

Photosensitizing agents activated by sunlight attracted more attention as a new generation of highly efficient and environmentally safe insecticide due to its rapid photo degradation in the visible light^18^. The present result showed that *Anopheles Pharoensis*, in different immature stages, were highly sensitive to the tested photosensitizers rose bengal and methylene blue, with 20 min. of sun light irradiation. Methylene blue exhibited privileged activity against *Anopheles pharoensis* different immature stages than rose Bengal.

Results revealed also that for photosensitizers to be active, an exposure to a visible source of light must happen, which takes place in this study using natural sunlight; this comes along with the recommendation by Khater and Hendawy^19^ of using sunlight instead of a light source. Methylene blue can be photoactivated with light sources of a range 630–700 nm, while light sources ranging from 380 to 520 nm are used to trigger the photosensitization reaction of rose bengal; both light sources are in the visible spectrum of sunlight. The previous photosensitizers introduce phototoxic effects by singlet oxygen (^1^O_2_) generation through a Type-II mechanism (excited triplet state photosensitiser (3Psen*) directly reacts with ground state triplet molecular oxygen (3O2), which is chemically reactive due to the presence of unpaired valence electrons^20^. These highly cytotoxic singlet oxygen molecules initiate multisite attacks against the intracellular proteins and cellular membranes in cells^21^. Singlet oxygen (^1^O_2_) is highly activated form of oxygen, it is not as stable as the ground state oxygen and will change back quickly (less than 0.04 microseconds) but in that time it will have affected its surrounding molecules by transferring its excitation energy to them and then returns to the ground state^22^.

The results revealed that the activity of tested photosensitizers is coupled with previous results recorded by Dondji et al.^23^, where different photosensitizers showed lethal effects to *Aedes aegypti*,* An. Stephensi and Culex quinquefasciatus* 4th larval instar, depending on the presence of light; however, rose bengal seemed to be more efficient at even lower concentrations than other photosensitizers against *Ae. aegypti* larvae, **Azizullah** et al.^24^ who recorded that chlorophyll derivatives, natural photosensitizers, can effectively be used against *Ae. aegypti* larvae, de Souza et al.^25^, where porphyrin (Photogem) in the presence of sunlight and fluorescent lamp recorded about 100.0% mortality in *Ae. aegypti* 2nd larval instar after 24 h, El-Shourbagy et al.^26^, where rose Bengal was the most effective dye against *Cu. pipiens* 4th larval instar followed by phloxine B, then rhodamine B, and El-Mehdawy et al.^14^, where rose bengal recorded LC_50_ and LC_90_ of 1.07, 1.19, 1.35, 1.65 µM, and 3.88, 3.98, 4.19, 4.51 µM against *Cu. pipiens* third larval instar, while methylene blue recorded LC_50_ and LC_90_ of 2.70, 2.79, 2.99, 3.08 µM and 4.82, 4.90, 5.04, 5.22 µM, respectively.

On the other hand, the results showed an enzymatic response to both photosensitizers depending on the targeted stage. A depression in the acetylcholinesterase (AChE) level in *An. pharoensis* immature stages as compared with untreated groups was recorded. For rose bengal, acetylcholinesterase (AChE) activity increased progressively for pupae at 24 h. As a biomarker of exposure to certain classes of pollutants, AChE activity measurements have become commonplace^27^. In addition, an elevated glutathione-S-transferase (GST) level in *The immature stages of An. Pharoensis were recorded by testing the photosensitizers in extracts as compared with the* control. The GST activity levels rose in larvae I than pupae stage in the same period. Biotransformation of foreign chemicals, drug metabolism, and protection from oxidative damage are all aided by GST^28^.

Molecular docking was performed for further evaluation of the role of both methylene blue and rose Bengal, which were investigated by applying the target molecules against AChE (PDB ID: 6xyu) through MOE 2015 apps. The observed results suggested that the stronger the contact and the greater the negative binding energy, the accurate the molecular docking analyses^29,30^. So, molecular docking validation can help in identifying potential photosensitizer agents’ targets for mosquito management. Molecular docking studies suggested that the acetylcholine receptor (AChR), Acetylcholinesterase (AChE) on *Aedes aegypti* mosquito larvae were potential targets of compounds containing 2-methyl-3,4-dihydroquinazolin-4-one heterocycle and were supported by bioactivity mechanisms^31^. Also, Mansour et al.^32^ used molecular docking to control the 3rd and 4th instars of *Culex pipiens* larvae using four bacterial strains and confirmed the role of the produced metabolites as larvicidal agents and Acetylcholine esterase inhibition. According to Ghaffar et al.^33^, the selected proteins after extensive silico molecular docking analyses had the potential to be an effective candidate for affecting the mortality of the larvae of *Tribolium castaneum.*

## Conclusion

This study provides significant insights into the efficacy of rose Bengal and methylene blue as biocidal agents against *Anopheles pharoensis* at various developmental stages. Both photosensitizers exhibited strong mortality rates, particularly at higher concentrations, with methylene blue showing superior toxicity across all stages compared to rose Bengal. The study also highlighted the progressive enzymatic responses, with both acetylcholinesterase and glutathione S-transferase activities increasing as the larvae matured, suggesting a compensatory detoxification mechanism. Molecular docking further confirmed the strong binding affinity of both photosensitizers to acetylcholinesterase, supporting their potential as effective control agents for mosquito populations. These findings underscore the promising application of photosensitizers in integrated pest management strategies for combating *Anopheles pharoensis* and possibly other mosquito vectors.

## Data Availability

The newly obtained sequence of Anopheles pharoensis has been bank-it to gene bank [https://www.ncbi.nlm.nih.gov/] and given accession number (PQ346929).
